# *Enterocytozoon bieneusi* Infection after Hematopoietic Stem Cell Transplant in Child, Argentina

**DOI:** 10.3201/eid3003.231580

**Published:** 2024-03

**Authors:** Cristian Javier Mena, Magalí Pérez Garófalo, Juliana Perazzo, Carolina Epelbaum, Gonzalo Castro, Paola Sicilia, Andrés Barnes, Lorena Guasconi, Verónica L. Burstein, Ignacio Beccacece, Mariel A. Almeida, Laura Cervi, Monica Santin, Laura S. Chiapello

**Affiliations:** National University of Córdoba Faculty of Chemical Sciences, Center for Research in Clinical Biochemistry and Immunology, Córdoba, Argentina (C.J. Mena, L. Guasconi, V.L. Burstein, I. Beccacece, M.A. Almeida, L. Cervi, L.S. Chiapello);; Dr. Juan P. Garrahan Pediatric Hospital, Buenos Aires, Argentina (M. Pérez Garófalo, J. Perazzo, C. Epelbaum);; Central Laboratory of the Province of Córdoba, Córdoba (G. Castro, P. Sicilia);; Microbiology Laboratory, Complementary Diagnostic Area, Hospital Rawson, Córdoba (A. Barnes);; USDA Agricultural Research Service, Beltsville, Maryland, USA (M. Santin)

**Keywords:** *Enterocytozoon bieneusi*, hematopoietic stem cell transplant, pediatric, Argentina, microsporidia, *Enterocytozoon bieneusi*-genotype D, parasites

## Abstract

We report a case of *Enterocytozoon bieneusi* infection in a pediatric hematopoietic stem cell transplant recipient in Argentina. Spores were visualized in feces using Calcofluor White and modified trichrome stainings. PCR and sequencing identified *E. bieneusi* genotype D in fecal samples and liver samples, confirming extraintestinal dissemination of the parasite.

Microsporidia, fungal-related single-cell parasites, infect a broad range of vertebrates and invertebrates. The most identified species of Microsporidia in humans are *Enterocytozoon bieneusi* and *Encephalitozoon intestinalis*, which have emerged as opportunistic pathogens in immunosuppressed persons, such as those infected with HIV, organ transplant recipients, and cancer patients. The infective forms of these parasites are the resistant spores that persist in the environment, causing infections through direct contact with infected persons, infected animals, or ingestion of contaminated water and food (*1*). Human microsporidiosis is characterized primarily by chronic diarrhea and wasting, with less frequent occurrences of extraintestinal disseminated disease. Identification to the genus and species level is crucial for tailored treatments, especially in cases of chronic diarrhea (*2*).

Pediatric patients undergoing allogeneic hematologic stem cell transplantation (HSCT) may experience gut-localized or extraintestinal microsporidiosis by *Encephalitozoon* spp (*3*). In patients with leukemia or lymphoma who receive cytotoxic treatments, intestinal infections are predominantly associated with *E. bieneusi*, and rare cases of extraintestinal dissemination also have been reported (*1*,*4*).

More than 500 worldwide genotypes of *E. bieneusi* have been identified based on genetic polymorphisms in the internal transcribed spacer of the rRNA gene. They are distributed into 11 distinct phylogenetic groups, with groups 1 and 2 comprising genotypes with zoonotic potential that infect humans and various mammalian and avian species (*2*).

Although intestinal microsporidiosis is prevalent in children residing in developing countries, scarce studies have been reported in Argentina (*1*,*5*,*6*). We present a case of *E. bieneusi* (genotype D) infection in a child who underwent unrelated allogeneic HSCT in Buenos Aires, Argentina. 

## The Study

A 12-year-old boy from Buenos Aires who had a January 2018 diagnosis of intermediate-risk pre-B acute lymphoblastic leukemia received an unrelated allogeneic HSCT in February 2022. A month after HSCT, the child was treated with antiviral therapy for reactivation of cytomegalovirus, adenovirus, and Epstein-Barr virus infections. Three months post-HSCT, under immunosuppressive therapy with tacrolimus (0.1 mg/kg/d), he received antimicrobial treatment with meropenem (60 mg/kg/d), linezolid (30 mg/kg/d), and liposomal amphotericin B (3 mg/kg/d) to combat prolonged fever and abdominal symptoms. Videoendoscopy of the upper digestive tract confirmed gastrointestinal graft-versus-host disease, and ultrasound showed splenomegaly with multiple rounded hypodense images in the spleen and liver. We also noted distension of the ileal and colonic loops, predominantly in the right colon, and ascites.

We treated the child with liposomal amphotericin B (3 mg/kg/d) to address persistent febrile symptoms and visceral lesions compatible with chronic disseminated candidiasis. Four months after HSCT, the child sought treatment for chronic diarrhea (>1 month) and abdominal pain. Prior to microbiological documentation, we prescribed empirical treatment of metronidazole (30 mg/kg/d), which produced no improvement of symptoms. 

Coproanalysis revealed typical polymicrobial bacterial flora, with no detection of bacterial toxins, adenovirus, rotavirus, or parasites. Calcofluor White and Weber’s modified trichrome staining revealed structures compatible with microsporidian spores in single and serial fecal specimens (Figure, panels A, B). Analysis of liver aspiration biopsy samples rendered no conclusive results. On the basis of microscopic results, we immediately initiated albendazole treatment (400 mg/d) for microsporidiosis (*7*).

**Figure:**
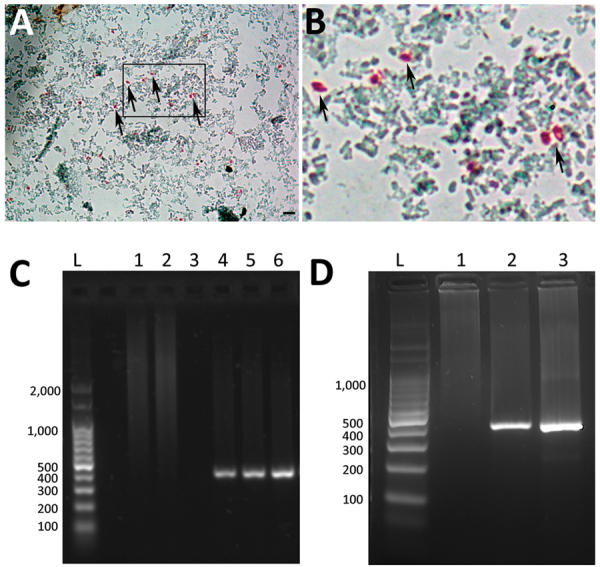
*Enterocytozoon bieneusi* detection in fecal sample and liver aspiration biopsy sample from a child with hematopoietic stem cell transplant, Argentina. A) Light microscopy of fecal samples after Weber’s modified trichome staining showing ovoid shaped-spores with a pinkish-red stained wall (arrows). Original magnification × 1,000; scale bars = 5 µm. B) Closer view of boxed area in panel A, showing spores (arrows). C) Agarose gel electrophoresis (2%) showing amplification products (≈390 pb) from nested PCR with inner primers EBITS1 and EBITS2 from patient fecal sample. Lane L, molecular weight ladder; lane 1, negative control (water); lanes 2 and 3, fecal samples from healthy donors; lane 4, positive feces control for *E. bieneusi*; lanes 5 and 6, fecal samples from patient. D) Nested PCR products from liver aspiration biopsy sample. Lane L, molecular weight ladder; lane 1, fecal sample from healthy donor; lane 2, fecal sample from patient; lane 3, liver aspiration biopsy sample from patient.

We conducted molecular biology studies based on fecal samples and liver aspiration biopsy samples. We determined *E. bieneusi* and genotype identification by using a nested PCR protocol that targeted the entire internal transcribed spacer and also amplified portions of the flanking large and small subunits of the ribosomal RNA (≈400 bp) gene (*8*,*9*) (Figure, panels C, D). We confirmed the presence of *E. bieneusi* genotype D based on Sanger sequencing using the inner-nested PCR primers (*2*). We named the nucleotide sequence generated BsAs1 and deposited it into GenBank (accession no. OP650902). Despite a decrease in diarrhea symptoms, the child died 18 days after initiation of albendazole treatment due to fulminant hyperacute lymphoproliferative syndrome, before identification of *E. bieneusi* was determined.

## Conclusions

*E. bieneusi* has been reported commonly in cancer patients undergoing chemotherapy (*1*,*3*,*4*,*10*). We report a case of *E. bieneusi* genotype D microsporidiosis, with intestinal and hepatic localization, in a child with leukemia and immunosuppression after a bone marrow transplant in Argentina. Our findings highlight the need to incorporate microsporidiosis in the differential diagnosis of immunosuppressed children after transplant surgery, as well as for other patient populations at high risk for opportunistic infections. Our report also emphasizes the critical importance of microsporidia identification because albendazole is effective against some *Encephalitozoon* species but not against *E. bieneusi* (*1*,*7*). Genotyping isolates of clinical *E. bieneusi* may help to identify potential environmental sources. Although nitazoxanide could be used as an alternative treatment, fumagillin has a wider range of activity effectively targeting *E. bieneusi* (*7*). The unavailability of fumagillin for treating human infections in several countries, including Argentina, underscores the need for enhanced accessibility to microsporidia treatment options, especially for vulnerable populations. 
